# On compromising with vaccine-hesitant families

**DOI:** 10.3389/fped.2024.1462958

**Published:** 2024-12-12

**Authors:** Gyan Chetan Moorthy, Jeffrey Thomas Poomkudy, Jennifer Walter

**Affiliations:** ^1^ Perelman School of Medicine, University of Pennsylvania, Philadelphia, PA, United States; ^2^ Feinberg School of Medicine, Northwestern University, Chicago, IL, United States; ^3^ Department of Medical Ethics, The Children's Hospital of Philadelphia, Philadelphia, PA, United States

**Keywords:** family centered care, vaccine hesistancy, public health, compromise, general pediatrics

## Introduction

My patient was a smiling, unvaccinated 6-year-old boy. Halloween was around the corner, and we bonded over his Spider-Man costume. When we got to vaccines, however, the mood expectedly soured. They were “unnatural,” his parents shared; the pharmaceutical industry was replete with liars and the CDC was bought. The only indication of progress since last visit's counseling was a new openness to the tetanus vaccine. They promised to think hard about it for next time but offered no explanation for why it was special. Perhaps they had a friend whose child fell sick with tetanus. I have often wondered if I should have dug more into it. What if I could have identified other vaccines that met their criteria?

## How should pediatricians respond to vaccine hesitancy?

When it comes to persuading vaccine-hesitant families to vaccinate, pediatricians are understandably expected to do the legwork. Their offices administer vaccines, and the relationships they build with families, sometimes spanning generations, position them to address individual concerns and bridge to the broader medical establishment. The best option is for pediatricians to attempt to convince parents about the benefits of vaccination. There are evidence-based recommendations for how to most effectively communicate about vaccine recommendations, including using closed-ended statements, bundling discussions of multiple vaccines and giving regular affirmation as part of broader motivational interviewing (1, 2).

However, when best practices fail, some pediatricians consider dismissing vaccine-hesitant families from their practice. While this may be acceptable in extreme circumstances (3), it interrupts care continuity and arguably subverts professional values like tolerance that are central to caring for patients in a pluralistic society (4). Some have argued that child welfare services should become involved to engage the courts to compel immunization. However, vaccine refusal generally fails legal and ethical standards for overriding parental authority to make health-related decisions for their child.

Weighing reliable protection from serious illness against relatively low risks for adverse health events, vaccination is widely regarded to be in a child's best interest. However, failure to act to *maximally* promote a child's interest is not sufficient to justify state intervention according to most interpretations of the best-interests standard (5). Moreover, state investigations can be disruptive and traumatizing, and the result in these cases is likely only to diminish trust in medical and governmental institutions. The harm principle and constrained parental autonomy model define stricter standards still (6, 7). They require that the vaccine be highly efficacious with low morbidity and that not receiving it put the patient at serious risk of imminent harm, or according to the constrained parental autonomy model, deprive a child of their basic needs. Partly due to herd immunity, which protects the individual unvaccinated child, the standards are not met. The pediatrician treating a child in a persistently vaccine-resistant family is left with few good options; they may eventually decide to ignore the topic altogether. But what if instead they offered to compromise with those families on when or even which vaccines their child receives, working with them to develop a schedule that integrates their values and preferences with pressing public health realities?

## Is compromise ethical?

The idea of any compromise on childhood vaccination understandably invites backlash. The CDC schedule reflects decades of careful study into immune system development on the one hand and infection spread patterns on the other. We know vaccination per the CDC schedule is safe and effective, but we do not know that for unofficial schedules. Some countries have experimented with prioritizing vaccines through selective mandates in response to outbreaks, but testing the effectiveness of these interventions is challenging in the short term, and there may even be decreased uptake of other vaccines (8). Responding to concerns about the quantity of shots administered in a single day, some pediatricians have published their own versions of the vaccine schedule, correctly condemned by experts as the substitution of limited experience for mountains of data. To our knowledge, none of these has been thoroughly studied. However, if some vaccination provides more protection than no vaccination—which follows from any individual vaccine efficacy study demonstrating protection against the specific disease as a health benefit —then it seems in the best interest of the child to receive even a reduced number of vaccines.

Pediatricians have a responsibility to promote public health, but their principal responsibility is to the children they treat. So, after failing to convince parents to vaccinate per the standard CDC or catch-up schedule, a pediatrician might, for example, emphasize vaccination of the child living in an older home against tetanus, for which there can be no herd immunity because spread is not from person to person and spend less energy on vaccination against hepatitis A, which is associated with self-limited illness and low rates of mortality. In so doing, this pediatrician is not claiming they know better than the CDC. Rather, they are engaging in a form of harm reduction, a strategy that has proved extremely effective in other areas of public health concern, such as substance use treatment (9). Harm reduction is aimed at minimizing the consequences of health-adverse choices when eliminating them is not immediately possible.

For families who reject the CDC schedule and have not responded positively to counselling, compromising on when, which, and how many vaccines to receive, may lead to partial immunity. This benefits both the child and their peers and prevents further estrangement from social institutions. Compromise as an approach is humble and non-coercive. It establishes an open, non-judgmental atmosphere that preserves the possibility for complete immunization, perhaps according to the evidence-based schedule, down the road. Had I asked the family of that costumed 6-year-old why they were reconsidering tetanus vaccination, I might have learned they were receiving health information from a new source or that their new priest had offered a vaccine-friendlier interpretation of scripture. I might have learned their neighbor knew someone who had gotten tetanus and nearly died. This could have been an opening to build additional rapport or focus on specific scientific misconceptions. Revisiting the conversation at the next visit with these reference points might have made the family that much more amenable to accepting vaccination, tetanus or otherwise. Building this shared understanding might be even more crucial if there were a future local outbreak of a highly communicable disease.

Some families have more nonspecific concerns about vaccines, informed by a mix of historic distrust of medical institutions due to discriminatory treatment, cultural preferences for traditional healing, peer anecdotes, and online misinformation. Other vaccine-hesitant families may have more concrete pseudoscientific beliefs, e.g., concerns about heavy metals or autism, and they may request a vaccination schedule that accommodates these heterodox beliefs. We recognize that by accepting an alternate timeline, there is a risk of validating those pseudoscientific beliefs. For this reason, it is important to be clear during counseling—which will have first aimed to correct any scientific misconceptions—that any alternate schedule is a compromise for the sake of a commonly desired end and not simply one of many equally defensible options.

## Discussion: what does ethical compromise look like, practically?

Primary care physicians must weigh several factors when customizing a vaccine schedule. One is the likelihood a disease will kill or seriously harm the patient, acknowledging that presentations can vary dramatically: sometimes polio is mild, and sometimes chicken pox is deadly. Another is the existence of treatment for the disease, and the patient's ability and willingness to access it, as well as the risks associated with treatment. Next, there is the risk profile of the vaccine itself. Then, there is the matter of what diseases are actively spreading in the patient's community. Finally, the physician must consider what pathogens would most threaten public health, including accounting for the patient's immunosuppressed contacts and how else they spend their time. For example, measles kills 2 in 1,000 children who catch it, has a 90 percent transmission rate, and is actively spreading across the United States.

Accurately performing this analysis for each patient is clearly a tall order – hence the CDC's schedule. However, with continued objection to vaccines, a physician should feel empowered to promote vaccines the family is likely to agree to, based on their stated objections, and recommend vaccines according to their good-faith estimate of medical usefulness. Pediatricians cannot imply that these customized vaccination suggestions are a substitute for evidence-based schedules. They must be offered as last-resort compromises, intended to avoid further alienating vaccine-hesitant families. Pediatricians who prefer not to accommodate an alternate schedule risk a missed opportunity to partially vaccinate a child, and parents might shop for more accommodating, but overall less scrupulous, providers.

Tailored vaccine counseling will require more time, energy, and expertise than most physicians have. There may be a unique role for the pediatric infectious disease specialist and local epidemiologists within this framework. Whether independently or as part of local public health data monitoring committees, they might be needed in consultation to support complex individual assessments and to compile and publish data about local outbreaks, community demographic and comorbidity data, and rates of vaccine uptake. This data could be integrated into an interactive tool to estimate semi-individualized likelihoods of illness susceptibility that could subsequently aid physicians making these complex compromises. The tool could be compared to a hospital antibiogram, which summarizes the susceptibility of bacteria cultured from patients to different antibiotics to inform antibiotic selection.

There is evidence to suggest some pediatricians already feel a level of comfort using an alternative immunization schedule upon parent request. Many also prioritize certain shots, like DTaP (diphtheria-tetanus toxoids-acellular pertussis) and the pneumococcal conjugate vaccine, both of which prevent diseases associated with serious morbidity and mortality in childhood, over the COVID-19 or influenza vaccines (10). Our hope is that expert infectious disease advice will help bring as much evidence as is reasonably possible to clinician *ad hoc* judgments and allow willing pediatricians to take a more active role in shaping a vaccine-schedule compromise, building and maintaining the durable, trusting connections with families that are the precondition for delivering high quality, mutually satisfactory healthcare. Safeguarding public health in the 21st century requires combatting vaccine hesitancy. This may require the flexibility for harm reduction, which safeguards autonomy while preserving a commitment to good health values.
